# p53-mediated disruption of FIP200 function cause autophagic dysregulation in a SCA7 polyglutamine disease model

**DOI:** 10.1186/1750-1326-8-S1-P68

**Published:** 2013-10-04

**Authors:** Xin Yu, Andrés Muñoz Alarcón, Abiodun Ajayi, Kristin Webling, Anne Steinhof, Ülo Langel, Anna-Lena Ström

**Affiliations:** 1 Department of Neurochemistry/Stockholm University, Stockholm, Sweden

## Background

Spinocerebellar ataxia type 7 (SCA7) is one of nine neurodegenerative disorders caused by expanded polyglutamine domains [1]. Aggregation of the different polyglutamine (polyQ) expanded disease proteins have been linked to the toxicity in these disorders [1]. We and others have shown that polyQ expanded proteins can be degraded by autophagy [2], and pharmacological activation of this pathway has hence been suggested as a therapeutic approach for these disorders. However, lately increasing evidence indicating that autophagy dysfunction occurs in several neurodegenerative disorders has emerged and raised concerns regarding using stimulation of this pathway as a therapeutic approach [3]. The aim of this study was to determine if and by which molecular mechanism(s) the expanded SCA7 disease protein ataxin-7 (ATXN7) affects the autophagic process.

## Materials and methods

Inducible control and SCA7 models expressing full-length ATXN7 with 10 (FLQ10) or 65 glutamines (FLQ65) were used. The autophagic activity was analyzed by measuring the p62 level and by monitoring the LC3 levels in the presence or absence of the lysosomal inhibitor bafilomycin A. Protein aggregation was analyzed by the filter trap method and protein-protein interactions by co-immunoprecipitation and western blot. Cellular toxicity was determined by the WST-1 assay.

## Results

We found that mutant ATXN7 causes autophagic dysfunction by altering the cytoplasmic activity of p53 resulting in an increased p53-FIP200 interaction, co-aggregation of p53-FIP200 with mutant ATXN7 and ultimately disruption of the key autophagy regulating FIP200-ULK1 complex. Furthermore, we show that treatment with a p53 inhibitor, or a blocker of ATXN7 aggregation, can restore the soluble levels of FIP200, as well as increase the autophagic activity and reduce mutant ATXN7 toxicity.

## Conclusions

We have identified a novel p53-mediated mechanism by which aggregating polyQ disease proteins can disrupts autophagic activity and showed that this inhibition contributes to polyQ toxicity. An increased understanding of the molecular mechanism by which autophagy inhibition occurs in neurodegenerative disease is of importance if safe and efficient therapeutic approaches based on autophagy stimulation should be developed.
